# *BVES* downregulation in non-syndromic tetralogy of fallot is associated with ventricular outflow tract stenosis

**DOI:** 10.1038/s41598-020-70806-4

**Published:** 2020-08-25

**Authors:** Yan Shi, Yongqing Li, Yuequn Wang, Ping Zhu, Yu Chen, Heng Wang, Shusheng Yue, Xiaohui Xia, Jimei Chen, Zhigang Jiang, Chengbin Zhou, Wanwan Cai, Haiyun Yuan, Yueheng Wu, Yongqi Wan, Xiaohong Li, Xiaolan Zhu, Zuoqiong Zhou, Guo Dai, Fang Li, Xiaoyang Mo, Xiangli Ye, Xiongwei Fan, Jian Zhuang, Xiushan Wu, Wuzhou Yuan

**Affiliations:** 1 The Center for Heart Development, State Key Lab of Development Biology of Freshwater Fish, Key Lab of MOE for Development Biology and Protein Chemistry, College of Life Sciences, Hunan Normal University, Changsha, 410081 Hunan China; 2 Guangdong Cardiovascular Institute, Guangdong Provincial People’s Hospital, Guangdong Academy of Medical Sciences, Guangzhou, 510100 Guangdong China

**Keywords:** Embryology, Disease model

## Abstract

BVES is a transmembrane protein, our previous work demonstrated that single nucleotide mutations of *BVES* in tetralogy of fallot (TOF) patients cause a downregulation of *BVES* transcription. However, the relationship between *BVES* and the pathogenesis of TOF has not been determined. Here we reported our research results about the relationship between *BVES* and the right ventricular outflow tract (RVOT) stenosis. *BVES* expression was significantly downregulated in most TOF samples compared with controls. The expression of the second heart field (SHF) regulatory network genes, including *NKX2.5*, *GATA4* and *HAND2*, was also decreased in the TOF samples. In zebrafish, *bves* knockdown resulted in looping defects and ventricular outflow tract (VOT) stenosis, which was mostly rescued by injecting *bves* mRNA. *bves* knockdown in zebrafish also decreased the expression of SHF genes, such as *nkx2.5*, *gata4* and *hand2*, consistent with the TOF samples` results. The dual-fluorescence reporter system analysis showed that *BVES* positively regulated the transcriptional activity of *GATA4*, *NKX2.5* and *HAND2* promoters. In zebrafish, *nkx2.5* mRNA partially rescued VOT stenosis caused by *bves* knockdown. These results indicate that *BVES* downregulation may be associated with RVOT stenosis of non-syndromic TOF, and *bves* is probably involved in the development of VOT in zebrafish.

## Introduction

Congenital heart disease (CHD) is the most common birth defect, exhibiting a mortality rate of more than 29%^1^. Within this group of conditions, the incidence of TOF is 7–10% in the USA^2^, 13.4% in Nigeria^3^, and 16% in India^4^. However, the aetiology of TOF has not been fully elucidated.

Anatomically, TOF has four distinct structural defects, namely, ventricular septal defect, overriding of the aorta, RVOT/pulmonary artery (PA) stenosis and right ventricular hypertrophy^5^. However, embryologically, the defects are thought to be caused by a single developmental error, involving the outflow tract funnel septum shifting left to right or forward, leading to a poor contraposition ventricular septal defect, overriding of the aorta and RVOT funnel stenosis^5–11^. RVOT stenosis eventually leads to right ventricular hypertrophy^10^. The degree of RVOT stenosis is a key clinical factor for the diagnosis of TOF^10^. However, the molecular mechanism behind RVOT/PA stenosis remains under investigation.

The development of the cardiac outflow tract has two major cell sources: one is neural crest cells, which provide cells for the distal development of the great artery by migration, while releasing signals to the SHF^7^; the other is anterior SHF^12^, and at the late stage of cardiac looping, cells from the anterior SHF are added to the outflow tract area, which promotes outflow tract elongation and the correct fusion of the outflow tract myocardial wall and ventricular septum^13^. Ablation of SHF may result in TOF due to an abnormal outflow tract^8^. In animal models, the knockout or knockdown of SHF regulatory network genes, such as *Gata4*^14^, *nkx2.5*^12,15^, *Tbx1*^16^, *Tbx20*^17^, and *Hand2*^18^, leads to abnormal development of the outflow tract. Functional mutations of SHF genes, such as *NKX2.5*^19–21^, *TBX1*^22,23^, *GATA4*^24,25^, *TBX20*^26^, and *HAND2*^27^, were found in TOF patients. The expression of these genes, including *NKX2.5*^28^, *GATA4*^28^, and *TBX1*^29^, was significantly decreased in the cardiac tissue samples of TOF, indicating that the downregulation of SHF genes is associated with outflow tract stenosis.

BVES, a blood vessel epicardial substance, also known as POPDC1, belongs to the Popeye domain containing (POPDC) gene family, encodes a novel class of cyclic adenosine monophosphate (cAMP) effector proteins, is a highly evolutionarily conserved membrane protein and is highly expressed in adult heart and skeletal muscle in vertebrates^30–32^. In mice, *Bves* is expressed in the development of cardiomyocytes^33^ and coronary endothelial cells^34^ and is highly expressed in the adult conduction system^35^. Knockout of *Bves* in mice was shown to cause sinus bradycardia under stress in an age-dependent manner^35^. In humans, *BVES* was found to be more highly expressed in ventricles than in atria^36^. Functional mutations of the *BVES* gene were detected in a family affected by hereditary muscular dystrophy with arrhythmia^37^, and in non-syndromic TOF^38,39^. It has also been shown that the expression of *BVES* was downregulated in patients with heart failure^36^ and non-syndromic TOF^39^. However, the relationship between *BVES* downregulation in non-syndromic TOF and outflow tract stenosis has not been determined.

Zebrafish have been used as an animal model for the development of VOT. In zebrafish embryos, SHF cells are derived from mesodermal *nkx2.5-* and *gata4*-positive cells^15^. Zebrafish *bves* expression began at the zygote transcript stage (1 h post fertilization, 1 hpf) and persisted through the developmental stages of the heart, including the SHF area and other cardiac tissues^40,41^. It was reported that the expression of the *Aggrecan* and *Cyp26* genes was changed in cardiac tissues with aortic valve disease and TOF^42,43^, and was also associated with the development of the cardiac outflow tract in zebrafish^42,44^. However, whether *bves* is involved in the development of VOT has not been determined.

In this study, *BVES* downregulation was detected in most of the 83 tissue samples from TOF patients together with the downregulation of the key SHF genes related to TOF. *bves* downregulation in zebrafish led to abnormal cardiac looping and VOT stenosis and subsequently led to downregulation of the key SHF genes. In addition, our findings suggested that *bves* and *nkx2.5* mRNA may partially rescue the VOT phenotype caused by the downregulation of *bves* to varying degrees.

## Materials and methods

All methods were performed in accordance with the relevant guidelines and regulations.

### Samples

This study was approved by the Ethics Committee of Hunan Normal University (NO. 014050), Guangdong General Hospital and the Institutional Ethics Committee of Guangdong Academy of Medical Sciences (GDREC2016186A). Written informed consent was obtained from each subject or their guardian.

TOF tissue samples were obtained from the hypertrophic muscle tissue of RVOT during open-heart surgery for TOF. Normal control samples were obtained from the RVOT tissue of individuals who died in accidents and agreed beforehand for their organs to be donated. Samples from cases with congenital heart-related diseases were excluded. Information about patients and normal controls is shown in Tables 1 and 2, respectively. After obtaining the tissue, it was quickly cut into small, 4–5 mm pieces with surgical scissors, placed into an Eppendorf tube, snap-frozen in liquid nitrogen and stored at − 80 °C for analysis. Zebrafish samples were selected under a microscope for embryonic development, while a embryo was selected as a sample for the extraction of RNA.

**Table 1:** Baseline information on the TOF samples.

Variables	Statistics (n = 83)
Male gender (%)	46 (54%)
Female gender (%)	38 (46%)
Age (years)	3.7 ± 8.9
Patent foramen ovale	56 (67%)
Atrial septal defect (%)	7 (8%)
Patent ductus arteriosus (%)	18 (22%)
Dextrocardia of aortic arch	1 (1%)
Congenital right aorta	4 (5%)
Venous anomalies (%)	6 (7%)
Coronary artery anomalies	3 (3%)
Endocardial cushion defect (%)	15 (18%)
Mesocardiac	1 (1%)
Other body defects	5 (6%)

Data are expressed as means and standard deviations, number or percentage. Venous anomalies contain persistent superior vena cava, inferior arch of innominate vein, persistent left superior vena cava into left atrium; Endocardial cushion defect contain tricuspid regurgitation, tricuspid insufficiency, mitral regurgitation, pulmonary regurgitation, pulmonary valve biology, pulmonary valve stenosis, absence of pulmonary valve.

**Table 2:** Baseline information of the control samples.

	Gender	Age	Cause of death	Other defects
CT1	Male	2 years	Drug abuse	No
CT2	Female	74 days	Encephalatrophy	No
CT3	Male	60 years	Encephalorrhagia	Hypertension

### Zebrafish lines

The AB strain of zebrafish was purchased from the Institute of Hydrobiology, Chinese Academy of Sciences. The transgenic line Tg (*cmlc2*:dsRed) was received as a gift from Didier Stainier, Max Planck Institute of Cardiovascular Research, Germany^45^, and Tg (*flia*:eGFP*)* from Qingshun Zhao, Nanjing Model Animal Research Center^46^. Adult zebrafish were raised and maintained under standard laboratory conditions^47,48^. The animal experimental protocol was approved by the Ethics Committee of Hunan Normal University (NO. 014050), and performed according to the relevant guidelines and regulations.

### Quantitative reverse-transcription polymerase chain reaction (qRT-PCR)

Total cDNA was prepared from whole embryos or tissues and qRT-PCR was performed as previously described^39^. In patient samples, the data of Ct values were normalized to *GAPHD*, and the fold change between normal control samples and TOF tissue samples was quantified using the 2^−ΔΔCT^ Livak Method. The significance was analyzed by Student’s *t*-test. In the same plate, the expression in patient tissue that was lower than the average expression of normal samples was defined as ‘downregulated’, while the expression in patient tissue was higher than the average expression of normal samples was defined as ‘upregulated’, and the data are presented as scatter points and histogram that were generated by GraphPad Prism 5. In zebrafish samples, the control group and knockdown group included at least three samples for each time point. The data of Ct values were normalized to *gapdh*, and the fold change between control group and knockdown group was quantified using the 2^−ΔΔCT^ Livak Method. The significance was analyzed by Student’s *t*-test. The data are presented in the form of a histogram that were generated by GraphPad Prism 5. All primer sequences are shown in Table S1.

### Plasmid construction

The construction of overexpression plasmid with pCMV-*BVES* and luciferase reporter plasmids with the human *NKX2.5*, *GATA4* and *MEF2C* promoters were performed as previously described^39^. Promotors of *TBX1*, *TBX20* and *HAND2* were amplified by primer 1 (forward, ATCGGTACCGAATTCagaatgtccaacacaacatcc; reverse, GTGATATCAGATCTCccatcaggcccagtctgagg, the capital letters are homologous arms on Vector), primer 2 (forward, ATCGGTACCGAATTCagaatgtccaacacaacatcc, reverse, GTGATATCAGATCTCccatcaggcccagtctgagg, the capital letters are homologous arms on Vector), and primer 3 (forward, ATCGGTACCGAATTCacacgagtaaggccggtttt, reverse, GTGATATCAGATCTCcggttagagctgtttggggt, the capital letters are homologous arms on Vector), repectively. pGL3-Bias plasmid were digested by restriction enzyme, *Xho*I, to linearize the plasmid. Promotor sequences were ligated into vectors using ClonExpress Ultra One Step Cloning Kit (Vazyme).

### Luciferase reporter assays

The luciferase reporter assays were analysesd as previously described^39^. HEK293T cell line was used, and the levels of firefly luciferase were standardised relative to that of *Renilla* luciferase.

### Morpholinos and mRNA injections

Zebrafish embryos were injected as reported previously^40^, and the sequences of the *bves* morpholino oligos was designed intended to block the translation of *bves* (5′-GATGTTGTGTTGGACATTCTGAGGC-3′, synthesised by GeneTools). pXT7-*bves* and pXT7-*nkx2.5* were linearized and used for in vitro transcription (Ribo m7G Cap Analogue, RiboMAX Large Scale RNA Production Systerm-T7; Promega). A total of 150 ng and 80 ng of capped mRNA of *bves* and *nkx2.5* was coinjected with *bves* morpholino, respectively.

### Phenotypic analyses of zebrafish

Embryos were incubated at 28.5 °C in petri dishes containing fresh water and maintained as described^48^. 6% methylcellulose was used to restrict the movement of zebrafish and Axiocam of Zeiss Company was used to photograph and analyse the morphology of juvenile fish at 48 hpf and 72 hpf. The heart phenotype of the juvenile fish at these two stages were analysed as previously described^48^. The range of motion of the heart was clearly seen, and the approximate position of VOT was observed. Under normal beating conditions of zebrafish heart, continuous photographs were taken with green fluorescence. Finally, the diastolic and systolic images were selected for analysis. Zeiss AxioVision 3.0.6 software and Adobe Photoshop were used to process images. We measured the width of ventricle outflow tract at the end-systolic stage using Digimizer, which normalised the scales added by Zeiss AxioVision 3.0.6 software automatically.

### Western blot

Total protein samples were prepared in radioimmunoprecipitation assay (RIPA) buffer, and the protein concentration was determined by BCA assay (Beyotime). Protein isolation was carried out in a 12% SDS polyacrylamide gel. Then, the protein was transferred to nitrocellulose membranes, blocked with 8% skim milk, and incubated with anti-BVES antibody (1:1,500 dilution; Absin) and anti-β-ACTIN antibody (1:3,000 dilution; Proteintech). The signal densities of BVES protein bands were quantified and normalised to β-ACTIN using ImageJ.

### Statistical analysis

The scatter points and histograms were generated by GraphPad Prism 5. To assess whether the experimental data of two groups and three groups were significantly different from each other, we applied Student’s *t*-test and one-way Anova, respectively. A *p* value of < 0.05 was considered statistically significant.

## Results

### Expression pattern of human *BVES* was related to pulmonary artery development

**Figure 1:**
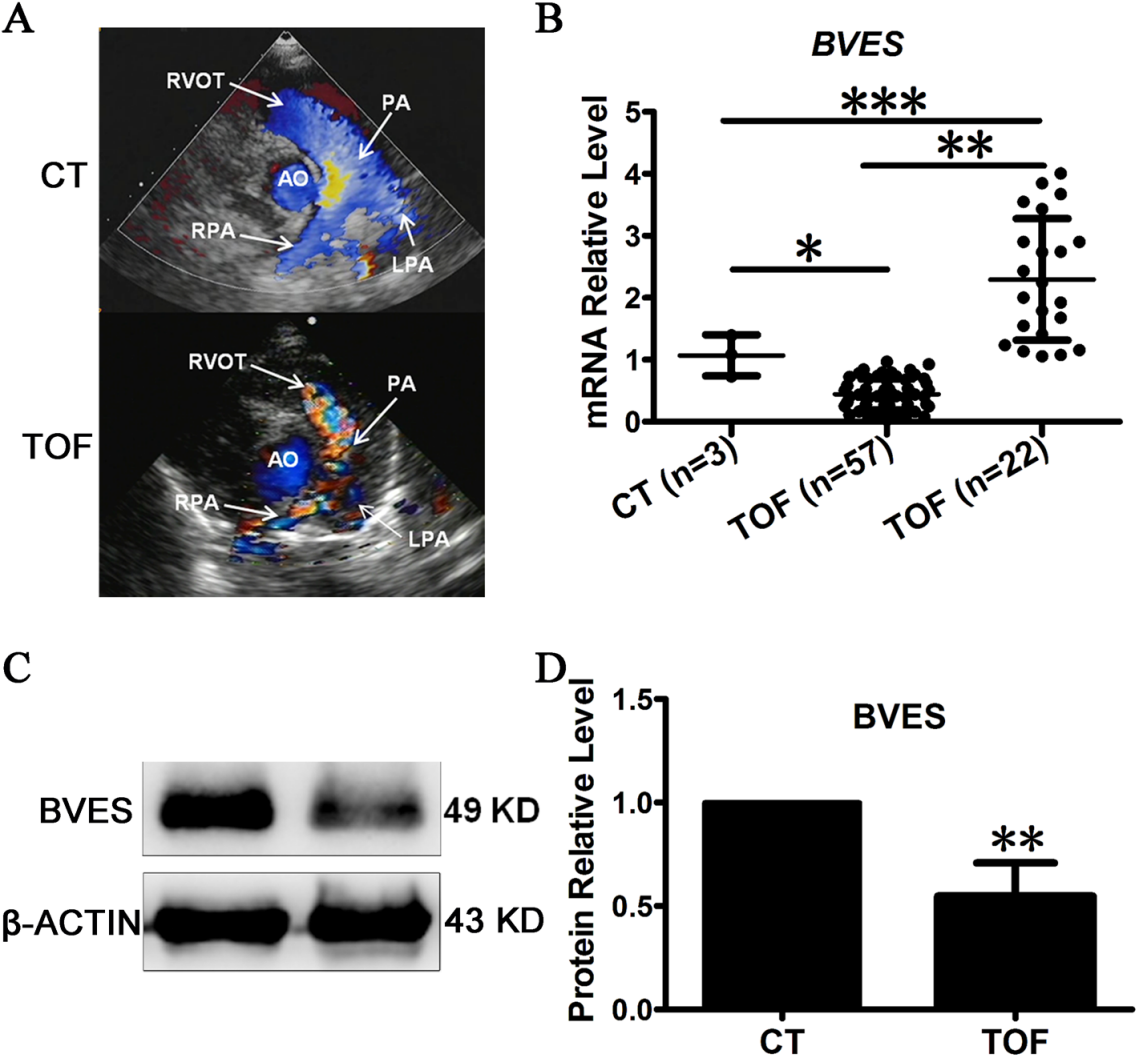
Relationship between the expression pattern of BVES and TOF. (**A**) The phenotype of RVOT stenosis in TOF diagnosed by echocardiography. RVOT, right ventricular outflow tract; AO, aorta; PA, pulmonary artery; RPA, right pulmonary artery; LPA, left pulmonary artery. CT, Control, the normal phenotype; TOF, tetralogy of fallot, the mal-phenotype of TOF patients. (**B**) qRT-PCR detected the expression of *BVES*. (**C**) Western blot detected the expression of BVES in downregulated samples. (**D**) Quantification of BVES by greyscale analysis. CT, control, RVOT tissue of normal controls; TOF, tetralogy of fallot, hypertrophic RVOT tissue of patients; n: number of samples. *p < 0.05; **p < 0.01; ***p < 0.001. The error bar shows the mean and SD.

The RVOT in TOF patients showed a phenotype of RVOT stenosis in all samples examined (Fig. 1A). The parasternal short axis view of cardiac colour Doppler echocardiography is important to assess the size of, for instance, the RVOT, PV, main pulmonary artery, and ascending aorta^49^. In normal people, the blood flow of RVOT and PA was antegrade and presented a single colour, while in TOF patients, it was aliasing and turbulent because of stenosis and presented multiple colours^50^ (Fig. 1A). The expression pattern of *BVES* in RVOT tissues with TOF was examined by qRT-PCR, and 79 of the 83 cases were valid for qRT-PCR analysis. Among them, the downregulation of *BVES* was detected in 57 cases, accounting for 72.2% of the total, while there was upregulation of *BVES* in the remaining 22 cases (Fig. 1B). Western blot analysis showed that the level of BVES protein expression in the three samples with *BVES* downregulation was approximately half that in the control (Fig. 1C,D). These results indicate that *BVES* downregulation could be related to TOF.

### Expression pattern of a set of SHF genes in RVOT stenosis with TOF

**Figure 2:**
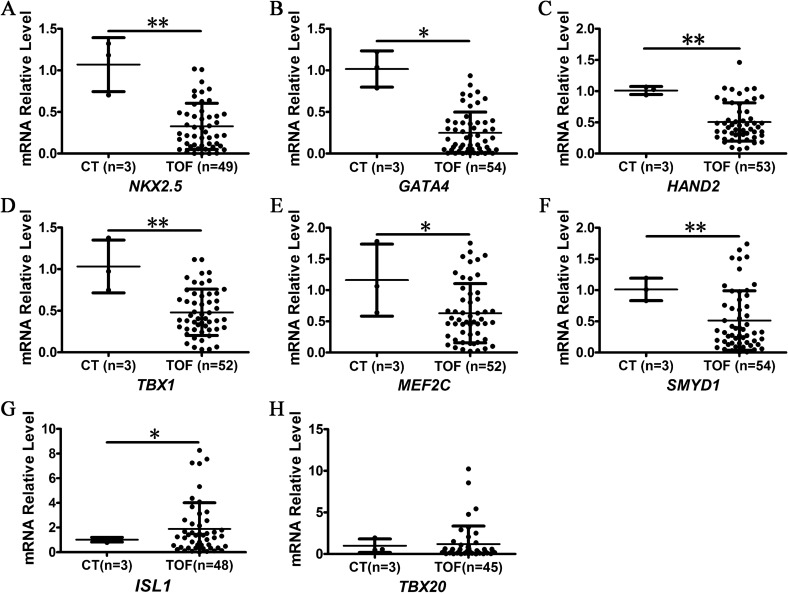
Detection of SHF gene expression. CT, control, RVOT tissue of normal controls; TOF, tetralogy of fallot, hypertrophic RVOT tissue of patients; n, Number of samples. *p < 0.05; **p < 0.01. The error bar shows the mean and SD.

It has been shown that the genes in the SHF regulatory network are required for the development of the cardiac outflow tract^2,5,6,9,10,51^. To examine the expression pattern of the SHF genes in RVOT stenosis tissue, the gene expression patterns in the RVOT tissues of TOF with *BVES* downregulation were analysed by qRT-PCR. The results showed that the expression of human *GATA4*, *NKX2.5*, *TBX1*, *HAND2*, *SMYD1* and *MEF2C* was significantly downregulated compared with that in the control (Fig. 2A–F), which is similar to the cases for *GATA4*, *NKX2.5* and *TBX1* observed in other studies^28,29^. Compared with the control samples, the mean expression of *ISL1* was higher than that of the control group, which was significant (Fig. 2G), although approximately 41.7% of the RVOT stenosis samples (20/48) were downregulated. However, the expression of *TBX20* was unexchanged (Fig. 2H). These results further indicate that the downregulation of the SHF genes is related to ROVT stenosis in TOF.

### *bves* knockdown in zebrafish led to abnormal heart looping

**Figure 3:**
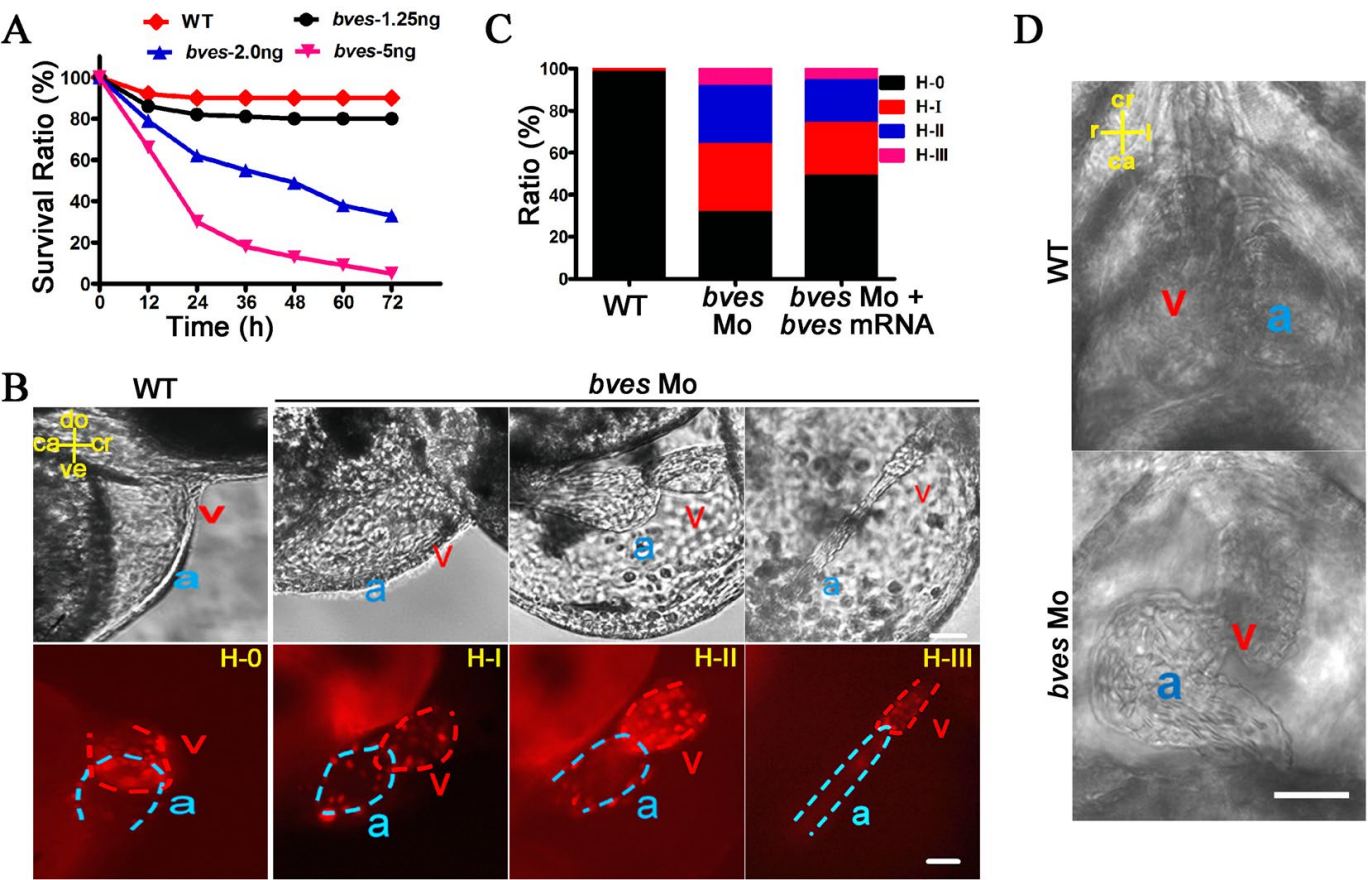
*bves* knockdown led to abnormal cardiac looping in zebrafish. (**A**) Survival ratio of wild type and morphants with 1.25, 2.0 and 5.0 ng of *bves* Mo at 72 hpf. WT, wild type; *bves*-1.25 ng, *bves* morphants with injection of 1.25 ng of *bves* Mo; *bves*-2.0 ng, *bves* morphants with injection of 2.0 ng of *bves* Mo; *bves*-5.0 ng, *bves* morphants with injection of 5.0 ng of *bves* Mo. (**B**) *bves* morphants showed cardiac defects at 48 hpf for the *cmlc2*:dsRed transgenic fish (a: atrium; v: ventricle), which were divided into four types based on the degree of cardiac dysplasia. H-0, the normal phenotypes; H-I, the moderate phenotypes; H-II, the strong phenotypes; H-III, the severe phenotypes. The position of the heart is marked by compass lines. ca, caudal; cr, cranial; do, dorsal; ve, ventral. Scale bar: 50 μm. (**C**) Data statistics of the different cardiac phenotypes in (**B**). (**D**) The morphants at 72 hpf with a left-looping heart in the ventral view. ca, caudal; cr, cranial; r, right; l, left. Scale bar: 50 μm. WT: wild type; *bves* Mo: *bves* morphants; *bves* Mo + *bves* mRNA, coinject *bves* morpholino and *bves* mRNA.

Previous studies showed that abnormal looping of the heart (the change of outer or inner curvature) disrupts the alignment between the ventricle and artery, leading to congenital heart phenotypes, such as TOF^8,52,53^. To explore the causal relationship in vivo between *BVES* downregulation in ROVT stenosis and the development of heart looping, a zebrafish *bves* knockdown model with *bves* morpholino was used. We chose a *bves* morpholino blocking *bves* translation that was reported in a previous report^40^. Knockdown efficiency was verified by analysing the survival rate of the morphants at 72 hpf. The results showed that the morphants with 2.0 ng were suitable for analysing the cardiac phenotype, which is consistent with a previous report^40^, while the morphants with 1.25 ng were similar to the wild type, and the morphants with 5 ng with a death occurred at 5 dpf (Fig. 3A). Therefore, a *bves* knockdown model, the morphants injected with 2.0 ng of morpholino were used in the subsequent analyses, we called it *bves* Mo.

To determine the effect of *bves* knockdown on cardiac development, the embryos of Tg (*cmlc2*:dsRed) zebrafish were used for *bves* morpholino injection, in which RFP was localized at the cardiomyocytes, facilitating live imaging of heart morphology. At 48 hpf, the morphants showed looping defects, cardiac dysplasia, and cardiac oedema (Fig. 3B). Classification into four phenotypes could be performed according to the degree of cardiac abnormality^48^, and approximately 70% of *bves* morphants showed defects of various degrees of severity, and this phenotype was partially rescued by coinjection with *bves* morpholino and *bves* mRNA (Fig. 3C). At 72 hpf, approximately 6.7% (3/45) of the morphants showed a left-looping heart in the ventral view (Fig. 3D), and the morphant with left-looping heart was not found in the WT or coinjection with *bves* morpholino and *bves* mRNA. These results indicated that knockdown of *bves* led to abnormal cardiac looping.

### *bves* knockdown in zebrafish causes VOT stenosis

**Figure 4:**
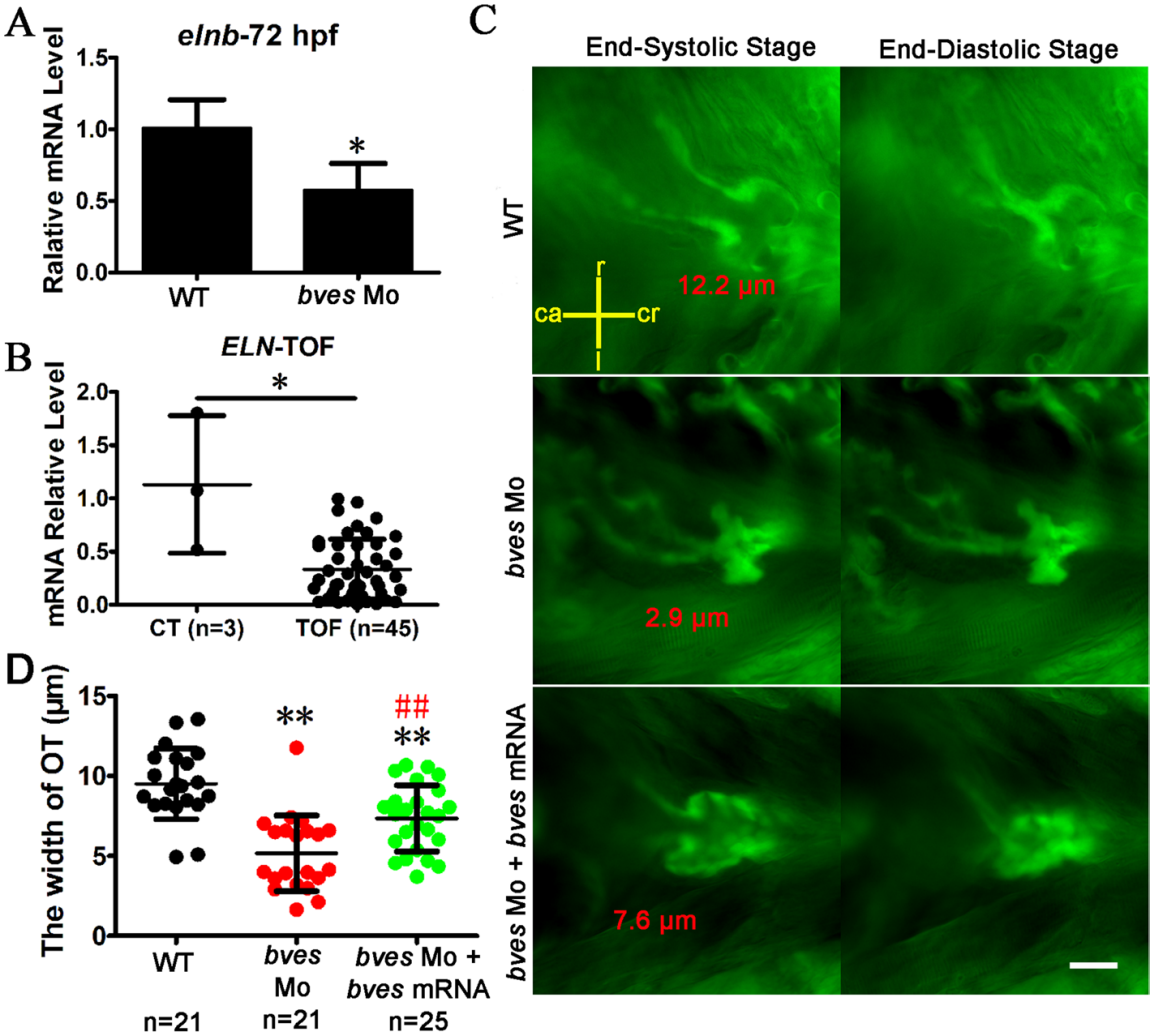
*bves* knockdown led to the phenotype of outflow tract stenosis in zebrafish with *flia*:*eGFP*. (**A**) qRT-PCR detected the expression of *elnb* in zebrafish at 72 hpf. WT, wild type; *bves* Mo, *bves* morphants. *p < 0.05. (**B**) qRT-PCR detected the expression of *ELN* in human tissue exapmles. CT, control, RVOT tissue of normal controls; TOF, tetralogy of fallot, hypertrophic RVOT tissue of patients. *p < 0.05. (**C**) *bves* morphants showed outflow tract defects at 72 hpf in Tg (*flia*:eGFP) zebrafish. The left column shows the outflow tract at the cardiac end-systolic stage, and the right column shows the end-diastolic stage. The words that marked by red color is the width of outflow tract. ca, caudal; cr, cranial; r, right; l, left. Scale bar = 15 μm. (**D**) Statistics about the width of outflow at the end-systolic stage in (**C**). Compared with the WT, the width of the outflow tract in *bves* knockdown mutants decreased by approximately 50% (WT, 9.5 μm; *bves* Mo, 5.0 μm, *p* < 0.01 compared with WT; *bves* Mo + *bves* mRNA, 7.5 μm, *p* < 0.01 compared with WT; *p* < 0.01 compared with *bves* Mo). OT, outflow tract; *Significance analysis with WT; ^#^Significance analysis with *bves* Mo. **p < 0.01; ^##^*p* < 0.01. n, number of samples. The error bar shows the mean and SD. *bves* Mo: *bves* morphants; WT: wild type; *bves* Mo + *bves* mRNA, coinject *bves* morpholino and *bves* mRNA.

The RVOT is the site of circulation between the right ventricular and the pulmonary artery^5,54^. *elnb*, a marker of the outflow tract in zebrafish, showed high expression at 72 hpf^55^. Compared with the wild type, the expression of *elnb* was downregulated in *bves* morphants (Fig. 4A), consistent with TOF patient samples (Fig. 4B). The results showed that, in the *bves* morphants, the VOT was abnormal. Stenosis of the PA or the RVOT is an indicator of TOF. To study the role of *bves* in the development of the outflow tract, the embryos of Tg (*flia*:eGFP) zebrafish were used for the *bves* morpholino injection, in which GFP is expressed in all endothelial cells^46^. In the wild type (Fig. 4C, top row), the VOT at 72 hpf was open at the end-systolic stage, while the VOT was closed at the end-diastolic stage (Fig. 4C). In the *bve*s morphants (Fig. 4C, middle row), the VOT was smaller and narrower than that in the wild type at the end-systolic stage. The width of VOT at the end-systolic stage was reduced approximately 50% (WT, 9.5 μm; *bves* Mo, 5.0 μm, *p* < 0.01) (Fig. 4D). The narrow phenotype was mostly rescued by *bves* mRNA (*bves* Mo + *bves* mRNA, 7.5 μm, *p* < 0.01 compared with WT; *p* < 0.01 compared with *bves* Mo) (Fig. 4C, bottom row; Fig. 4D). These results suggest that *bves* knockdown affected the development of outflow tract, leading to the phenotype of VOT stenosis.

### *bves* regulated VOT development via SHF genes

**Figure 5:**
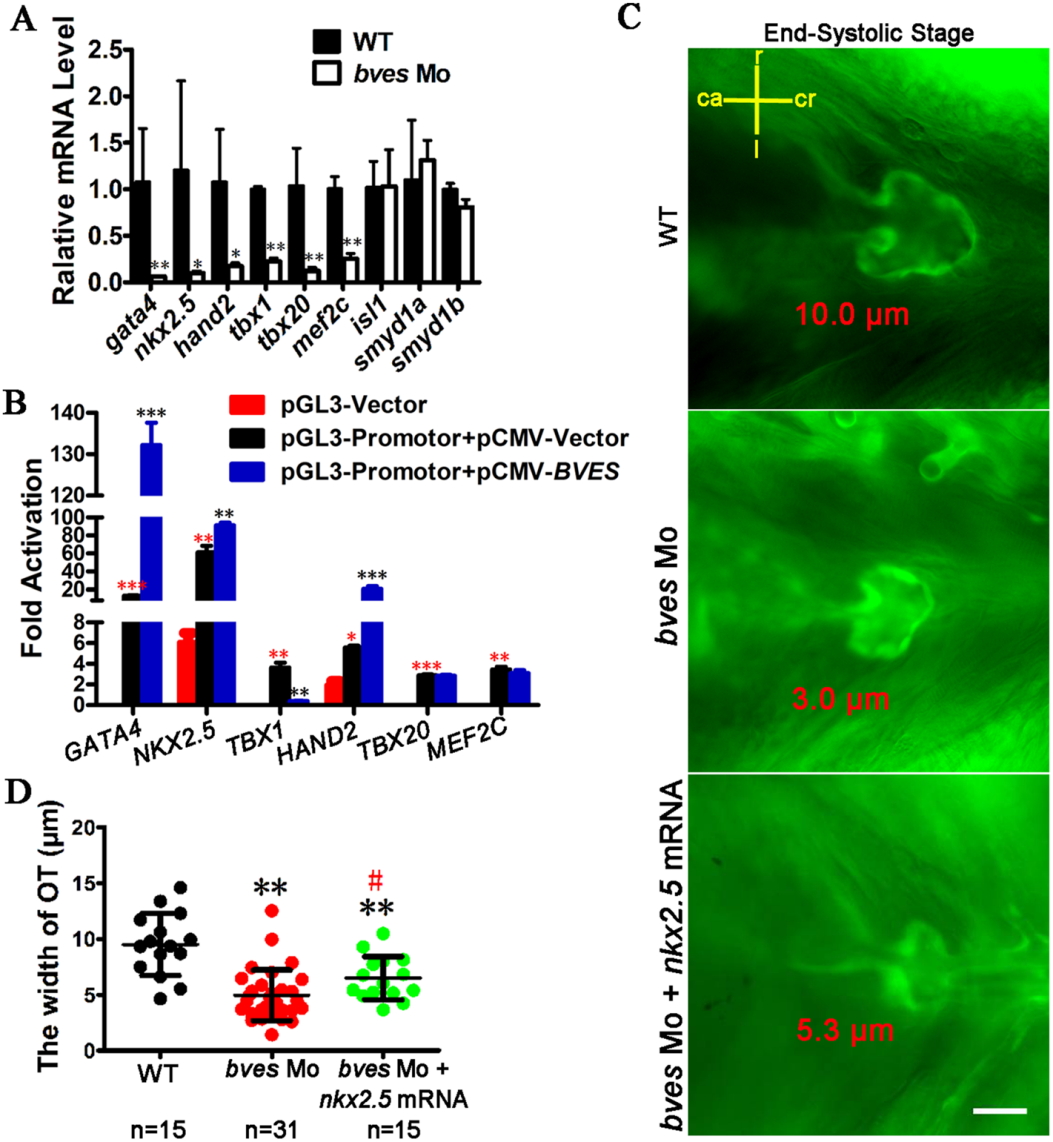
*bves* regulates the development of the outflow tract via SHF. (**A**) SHF gene expression was detected in *bves* morphants by qRT-PCR at 48 hpf. (**B**) Overexpression of *BVES* led to upregulation of transcriptional activity at the promoters of *GATA4*, *NKX2.5* and *HAND2*, downregulation of transcriptional activity at the promoters of *TBX1*, and no change in transcriptional activity at the promoters of *TBX20* and *MEF2C*. pCMV-Vector, empty vector of pCMV-Myc; pCMV-*BVES*, the vector overexpressing *BVES*; pGL3-Vector, empty vector of pGL3-Bias; pGL3-Promotor, vector containing the promotor of gene. Red *: significance analysis with pGL3-Vector group; Black *: significance analysis with pGL3-Promotor + pCMV-Vector group. *p < 0.05; **p < 0.01; ***p < 0.001. (**C**) Outflow tract phenotype of the wild type, *bves* Mo injected, *bves* Mo and *nkx2.5* mRNA coinjected in end-systolic at 72 hpf in Tg (*flia*:eGFP). The words that marked by red color is the width of outflow tract. ca, caudal; cr, cranial; r, right; l, left. Scale bar = 15 μm. (**D**) The width of the outflow tract at the end-systolic stage in (**C**). Compared with the WT and *bves* Mo, the width of the outflow tract was partially rescued by *nkx2.5* mRNA (WT, 9.5 μm, *bves* Mo, 5.0 μm, *p* < 0.01 compared with WT; *bves* Mo + *nkx2.5* mRNA, 6.3 μm, *p* < 0.01 compared with WT, *p* < 0.05 compared with *bves* Mo). n, number of samples. OT, outflow tract. *Significance analysis with WT; ^#^Significance analysis with *bves* Mo. ^#^*p* < 0.01; **p < 0.01. The error bar shows the mean and SD. WT: wild type; *bves* Mo: *bves* morphants; *bves* Mo + *nkx2.5* mRNA, coinject *bves* morpholino and *nkx2.5* mRNA.

The development of VOT at the late stage of cardiac looping is closely related to the SHF genes^2,5,6,9,10,51^. To detect the effects of SHF genes on the development of outflow tract, their expression in the *bves* morphants at 48 hpf was analysed by qRT-PCR. As seen in Fig. 5A, the expression of *gata4*, *nkx2.5*, *hand2*, *tbx1*, *tbx20* and *mef2c* was significantly downregulated, which is consistent with the downregulation detected in the RVOT tissues of TOF (Fig. 2), except for *tbx20*. The expression of *smayd1a*, *smayd1b* and *isl1* was unchanged (Fig. 5A). To study the regulatory relationship between *bves* and the SHF genes, *GATA4*, *NKX2.5*, *TBX1*, *HAND2*, *TBX20* and *MEF2C* were chosen for dual-fluorescence reporter system analysis. The results showed that the overexpression of *BVES* in the HEK293T cell line significantly increased the transcriptional activities of the promoters of *GATA4*, *NKX2.5* and *HAND2* by tenfold, 1.5-fold and threefold, respectively (Fig. 5B). For other transcriptional activities, *TBX1* was decreased by ninefold, and *TBX20* and *MEF2C* were unchanged. These results suggest that genes in the SHF regulatory network, such as *GATA4*, *NKX2.5* and *HAND2*, are potential factors involved downstream of *BVES*.

To further confirm that *bves* regulates VOT development through the SHF genes, we coinjected *bves* morpholino and *nkx2.5* mRNA into Tg (*flia*:EGFP) transgenic zebrafish and analysed the development of VOT at 72 hpf. The results showed that the width of VOT was partially rescued by *nkx2.5* mRNA (Fig. 5C,D) (WT, 9.5 μm, *bves* Mo, 5.0 μm, *p* < 0.01 compared with WT; *bves* Mo + *Nkx2.5* mRNA, 6.3 μm, *p* < 0.01 compared with WT, *p* < 0.05 compared with *bves* Mo). These results indicated that *bves* regulated the development of VOT via its downstream genes, such as *nkx2.5*, *gata4* and *hand2*. However, the results need to be further studied.

## Discussion

Our previous report showed that *BVES* allelic variants are associated with RVOT stenosis in TOF patients, which leads to the downregulation of *BVES* itself at the transcriptional and protein levels in the tissues of cases of RVOT stenosis with TOF containing allelic variants^38,39^. These results suggest that the downregulation caused by the *BVES* allelic variants was related to the phenotype of RVOT stenosis, consistent with the finding that gene downregulation is associated with RVOT stenosis, as demonstrated by other investigators^28,29,38^. However, the mechanism underlying the association between *BVES* downregulation and RVOT stenosis has not been elucidated. In this study, *BVES* downregulation was detected in approximately 72.2% of the samples (57/79) from cases of RVOT stenosis with TOF with decreases of both mRNA and protein levels by half. The results presented in this paper are consistent with a previous report^39^, suggesting that the downregulation of the *BVES* gene in RVOT stenosis with TOF is an aetiology, and not only the aetiology that lead to the phenotype of TOF.

It has been shown that, at the late stage of cardiac looping, cells from the anterior SHF are added to the outflow tract area for outflow tract elongation and the correct fusion of the outflow tract myocardial wall and ventricular septum^6,7,13^. Ablation of SHF may result in TOF due to an abnormal outflow tract^8^. In this study, compared with the normal RVOT samples, the expression of genes in the SHF regulatory network, such as *NKX2.5* and *GATA4*, *HAND2*, *TBX1*, *MEF2C* and *SMYD1*, were significantly decreased in the TOF samples, with *BVES* being mostly downregulation (Fig. 2) and occasionally upregulated (data unpublished). In animal models, knockout or knockdown of SHF regulatory network genes, such as *Gata4*^14^ and *nkx2.5*^12,15^, leads to abnormal outflow tract development. The expression of *NKX2.5* and *GATA4* in the open-heart surgery of CHD tissues is downregulated^28^, which is similar to that in the myocardial tissue at the earlier stage of embryonic development in the *nkx2.5* and *gata4* knockdown animal models^12,15^. These results suggest that the downregulation of gene expression detected in the tissues obtained during surgery may be similar to the gene downregulation in embryonic development. In our zebrafish model, knockdown of *bves* significantly decreased the expression of the five genes in the SHF regulatory network, including *nkx2.5*, *gata4*, *hand2*, *mef2c* and *tbx1* (Fig. 5A), suggesting that *bves* downregulation may be the cause of the maldevelopment of heart looping and VOT. The results with a dual-fluorescence reporter system confirmed that *BVES* positively regulated the transcriptional activity of *GATA4*, *NKX2.5* and *HAND2* and negatively regulated the transcriptional activity of *TBX1* promoters (Fig. 5B). The above results suggest that *GATA4*, *NKX2.5* and *HAND2* are potential downstream genes of the *BVES* gene.

*Nkx2.5* and *Gata4*, as marker of cardiac progenitors, together regulate the arrangement of ventricle outflow^14^. In *Gata4*+/− mice, a small number of heterozygote mutants were double outlet right ventricle (DORV), and in *Gata4*+*/−*;*Gata5*+/− double heterozygote mutants, almost all embryos were DORV, some of which had further aortic stenosis. *Hand2*, a heart and neural crest derivative, is important for the development of the heart, especially in the outflow tract^56^. In the mutant mice of *Hand2f/f;Mef2c*-*Anf*-*Cre*, which specifically deleted *Hand2* in the SHF progenitors, the OFT lumen was narrowed, and the OFT wall was thickened^57^. In other mutant mice of *Hand2f/-*;*Wnt1*-*Cre* cKO, which specifically deleted *Hand2* in the neural crest, a variety of arterial malformations, including pulmonary stenosis, were observed^18^. In this paper, *nkx2.5* mRNA only partially rescued the OFT malformation caused by *bves* Mo, which indicated that *bves* caused OFT abnormalities by widely regulating the heart development gene, not one of the heart development genes. The cells of outflow tract are different from anterior lateral plate mesoderm (ALPM), where SHF progenitors are specified in higher vertebrates. The descendants of *gata4*+ or *nkx2.5*+ cell at ALPM are crucial for the development of outflow tract by compromised progenitor cell proliferation^15,58,59^. *Nkx2.5* and *Gata4* play an important role in the outflow tract by regulating the SHF genes, but they also play a vital role in early specialization of cardiomyocyte progenitor cells^60,61^. In our *bves* knockdown model in zebrafish, the early heart development was affected (Fig. 3), so *bves* leading to the abnormal phenotypes of outflow tract may be affected both by the outflow tract development via regulating SHF genes and the indirect consequence of early defects via regulating early cardiac progenitor specification. However, the molecular regulatory mechanism between *bves* and *nkx2.5*, *gata4* and *hand2* requires further study.

BVES, encodes a transmembrane protein, that contains three transmembrane domains and a Popeye domain. The Popeye domain makes up a large part of the cytoplasmic portion of the protein and functions as a cAMP-binding domain^62,63^. In both mice and zebrafish, *Bves* act as effector proteins of cAMP, which control the development of the conduction system by combining ion channel genes^35,37^. Furthermore, *BVES* has a functional mutant in AV-block patients^37,64^. However, in the process of carcinogenesis, *BVES* inhibits epithelial–mesenchymal transition (EMT) and cell adhesion of cancer cells to prevent the formation of cancer via GEFT/Rho signalling and WNT signalling^65^. In our previous study, *BVES* also had functional polymorphisms in TOF patients^39^, but whether *BVES* is related to the maldevelopment of OFT has not been studied to date. In this paper, we illustrate that *bves* is related to the development of OFT in zebrafish, and the potential target genes of *bves* are *nkx2.5*, *gata4* and *hand2*. Whether *bves* acts as an effector protein of cAMP or as a signal transduction member, such as GEFT/Rho signalling and WNT signalling, to regulate the expression of *nkx2.5*, *gata4* and *hand2* needs further study.

Zebrafish, as an animal model, has been used to study the pathogenesis of many diseases due to its simple developmental structure of the organs studied. In terms of studies on cardiac diseases, zebrafish have been used in research on heart failure^66^, congenital heart defects^67,68^, and outflow tract disease^42,69^. In this paper, we used morpholinos to downregulate *bves* expression in zebrafish. The downregulation of *bves* in zebrafish caused looping defects, cardiac dysplasia, cardiac oedema (Fig. 3B), stenosis of the VOT (Fig. 4C) and several heart rate abnormalities (data unpublished). Abnormal heart rhythm leads to abnormal ventricular contraction, which makes the cilia of the developing heart and outflow tract feel different shear stress, thereby affecting the development of the heart and outflow tract^70,71^. In our *bves* knockdown model, we found that some of the mutants had abnormal rhythms. Because the phenotype of outflow tract stenosis also appears in the mutants with normal heart development and rhythm, we think that the difference in outflow tract dynamics caused by ventricular contract was considerably less than outflow tract malformation. However, further studies need to be performed to support this hypothesis. In addition, the abnormal phenotypes of VOT and heart looping were partially rescued by *bves* mRNA (Figs. 3D, 4C), supporting the hypothesis that the phenotypes were partially caused by the downregulation of *bves*, and several might be caused by the toxicity of the morpholino. To further explore the molecular mechanism by which *bves* induced developmental abnormalities in the heart and VOT, it is necessary to establish a *bves* knockout zebrafish line.

In summary, we studied the relationship between *BVES* downregulation and the occurrence of RVOT stenosis in TOF using human RVOT stenosis samples and a zebrafish model of *bves* downregulation. Our results show that *bves* is required for the development of VOT in zebrafish, suggesting that *BVES* downregulation is associated with the occurrence of RVOT stenosis in non-syndromic TOF patients.

## Supplementary information

Supplementary file1
