# Supplementary material for: BVES downregulation in non-syndromic tetralogy of fallot is associated with ventricular outflow tract stenosis
Source: Sci Rep. 2020 Aug 25;10:14167. doi: 10.1038/s41598-020-70806-4 (PMC7447802; doi:10.1038/s41598-020-70806-4)
Supplement: Supplementary file 1 — Supplementary file1 [file 41598_2020_70806_MOESM1_ESM.docx]

# Supplementary material

***BVES* Downregulation in Non-syndromic Tetralogy of Fallot is Associated with Ventricular Outflow Tract Stenosis**

Yan Shi^a,1^, Yongqing Li^a,1^, Yuequn Wang^a,1^, Ping Zhu^b,1^, Yu Chen^a^, Heng Wang^a^, Shusheng Yue^a^, Xiaohui Xia^a^, Jimei Chen^b^, Zhigang Jiang^a^, Chengbin Zhou^b^, Wanwan Cai^a^, Haiyun Yuan^b^, Guanren Wu^b^, Yongqi Wan^a^, Xiaohong Li^b^, Xiaolan Zhu^b^, Zuoqiong Zhou^b^, Guo Dai^a^, Fang Li^a^, Xiaoyang Mo^a^, Xiangling Ye^a^, Xiongwei Fan^a*^, Jian Zhuang^b,*^, Xiushan Wu^a,*^ and Wuzhou Yuan^a,*^

^a^The Center for Heart Development, State Key Lab of Development Biology of Freshwater Fish, Key Lab of MOE for Development Biology and Protein Chemistry, College of Life Sciences, Hunan Normal University, Changsha, Hunan, China

^b^Department of Cardiac Surgery, Guangdong Cardiovascular Institute, Guangdong General Hospital, Guangdong Academy of Medical Sciences, Guangzhou, Guangdong 510100, China

Table S1 qRT-PCR primers

| Gene | Primer | | Size | TM |
| --- | --- | --- | --- | --- |
|  | Forward | Reverse | (bp) | (℃) |
| *ISL1*-Human | ctgcttttcagcaactggtca | taggactggctaccatgctgt | 123 | 60 |
| *TBX1-Human* | cggtgaagaagaacgcgaag | ggatccatgccgaagagctt | 157 | 60 |
| *GAPDH*-Human | cgaccactttgtcaagctca | cccctcttcaaggggtctac | 127 | 60 |
| *SMYD1*-Human | ggtaacattgaggtggggca | gtagctccatctccgtctgc | 127 | 60 |
| *TBX20*-Human | tggacaacaagaggtaccgc | aatgatgtgcacccttggct | 239 | 60 |
| *BVES*-Human | ccactctctaccgatgtgcc | ccggtacatgccactgagtt | 137 | 60 |
| *GATA4*-Human | ctcctactccagcccctacc | gcggggtggacatagccc | 215 | 60 |
| *NKX2.5*-Human | ccctcctcctgcatgctg | tttcaggctttcttttcggctc | 215 | 60 |
| *MEF2C*-Human | gagccggacaaactcagaca | actggcatctcgaagttggg | 198 | 60 |
| *HAND2*-Human | cgcaggactcagagcatcaa | tcttgtcgttgctgctcact | 268 | 60 |
| *ELN*-Human | aggggttgtgtcaccagaag | cagctccaaccccgtaagta | 110 | 60 |
| *gapdh*- Zebrafish | atcatctctgccccaagtgc | acggtcttctgtgttgctgt | 206 | 60 |
| *isl1*-Zebrafish | ccagcctgctttccaacaac | ggactcgctaccatgctgtt | 126 | 60 |
| *smyd1a*-Zebrafish | tttcgccgctgttgtgttg | ccgatgtttctgatggcgga | 177 | 60 |
| *smyd1b*-Zebrafish | agctgacgactctggaggat | tgatcgctcaccatgaaccc | 187 | 60 |
| *tbx1*-Zebrafish | ccgaacaaaacgctggtgaa | tcaggagcatgtaatctgcca | 193 | 60 |
| *tbx20*-Zebrafish | ctcttccccaaaacctcagct | aggtttggatggcatgacga | 153 | 60 |
| *gata4*-Zebrafish | gtgcaatgcctgtggactct | gactggctctccttctgcat | 163 | 60 |
| *hand2*-Zebrafish | agaggccttcaaagcggaat | ccaatgcccaaacatgctg | 150 | 60 |
| *mef2c*-Zebrafish | gattgcgcggataatggacg | actttgtccatgtccgtgct | 174 | 60 |
| *nkx2.5*-Zebrafish | atgccatccggatcctctct | tcagatcttcacccgggtct | 207 | 60 |
| *bves*-Zebrafish | gcggagggtcacagatacag | cgtctccgcatgtgtttgg | 161 | 60 |
| *elnb*-Zebrafish | actggtggattaggcacagc | caacacctgtaccacctgct | 109 | 60 |
